# 3'-[^18^F]fluoro-3'-deoxythymidine ([^18^F]FLT) Positron Emission Tomography as an In Vivo Biomarker of inhibition of CDK 4/6-Rb pathway by Palbociclib in a patient derived bladder tumor

**DOI:** 10.1186/s12967-022-03580-8

**Published:** 2022-08-18

**Authors:** James L. Tatum, Joseph D. Kalen, Paula M. Jacobs, Lisa A. Riffle, Amy James, Lai Thang, Chelsea Sanders, Melinda G. Hollingshead, Falguni Basuli, Jianfeng Shi, James H. Doroshow

**Affiliations:** 1 Division of Cancer Treatment and Diagnosis, National Cancer Institute, National Institutes of Health, Bethesda, MD United States; 2 Small Animal Imaging Program, Laboratory Animal Sciences Program, Frederick National Laboratory for Cancer Research, Frederick, MD United States; 3 Animal Research Technical Support, Laboratory Animal Sciences Program, Frederick National Laboratory for Cancer Research, Frederick, MD United States; 4 Biological Testing Branch, Developmental Therapeutics Program, Division of Cancer Treatment and Diagnosis, National Cancer Institute, National Institute of Health, Frederick, MD United States; 5 Chemistry and Synthesis Center, National Heart, Lung, and Blood Institute, National Institutes of Health, Bethesda, MD United States

**Keywords:** Imaging biomarkers, Palbociclib, CDK4/6, FLT PET, Response biomarker

## Abstract

**Background:**

Several new generation CDK4/6 inhibitors have been developed and approved for breast cancer therapy in combination with endocrine therapeutics. Application of these inhibitors either alone or in combination in other solid tumors has been proposed, but no imaging biomarkers of response have been reported in non-breast cancer animal models. The purpose of this study was to evaluate 3'-[^18^F]fluoro-3'-deoxythymidine ([^18^F]FLT) Positron Emission Tomography (PET) as in vivo biomarker of response to palbociclib in a non-breast cancer model.

**Methods:**

Twenty-four NSG mice bearing patient derived xenografts (PDX) of a well-characterized bladder tumor were randomized into 4 treatment groups: vehicle (n = 6); palbociclib (n = 6); temozolomide (n = 6); and palbociclib plus temozolomide (n = 6) and treated with two cycles of therapy or vehicle. Tumor uptake of [^18^F]FLT was determined by micro-PET/CT at baseline, 3 days, and 9 days post initiation of therapy. Following the second cycle of therapy, the mice were maintained until their tumors reached a size requiring humane termination.

**Results:**

[^18^F]FLT uptake decreased significantly in the palbociclib and combination arms (p = 0.0423 and 0.0106 respectively at day 3 and 0.0012 and 0.0031 at day 9) with stable tumor volume. In the temozolomide arm [^18^F]FLT uptake increased with day 9 uptake significantly different than baseline (p = 0.0418) and progressive tumor growth was observed during the treatment phase. All groups exhibited progressive disease after day 22, 10 days following cessation of therapy.

**Conclusion:**

Significant decreases in [^18^F]FLT uptake as early as three days post initiation of therapy with palbociclib, alone or in combination with temozolomide, in this bladder cancer model correlates with an absence of tumor growth during therapy that persists until day 18 for the palbociclib group and day 22 for the combination group (6 days and 10 days) following cessation of therapy. These results support early modulation of [^18^F]FLT as an in vivo biomarker predictive of palbociclib therapy response in a non-breast cancer model.

**Supplementary Information:**

The online version contains supplementary material available at 10.1186/s12967-022-03580-8.

## Introduction

The D-cyclin-dependent kinase 4/6-INK4-retinoblastoma (D-CDK 4/6- Rb) pathway is a key pathway regulating the G1-S phase transition in the cell cycle. Abnormal activation of this pathway has been reported in numerous cancer types and it has been a high-profile target for therapeutic intervention, including high specificity inhibitors of the pathway [1, 2], which arrest tumor cells at G1. CDK 4/6 inhibitors are not new and have been employed effectively but were limited by dose toxicity. However, several new generation inhibitors have improved therapeutic indices and three, palbociclib, ribociclib, and abemaciclib, have been approved by FDA for the treatment of ER + /HER2- breast cancer in combination with endocrine therapy. [3].

Growing evidence supports expansion of these new CDK 4/6 inhibitors to other solid tumors [4], such as lung, melanoma, and head and neck cancers, with pre-clinical data showing response in human tumor xenografts. [5] Successful translation of a new therapy is highly dependent on robust biomarkers and a non-invasive response marker could be useful in translation of these inhibitors into other tumors. A recent preclinical study of six ER + breast cancer cell lines and one related xenograft reported that [^18^F]FLT and [^18^F]ISO-1 might be useful as predictive biomarkers for ER + breast cancer treated with the combination palbociclib/fulvestrant. [6] Another preclinical study [7] noted similar [^18^F]FLT responses in the presence of palbociclib in a triple negative breast cancer model with protein pRB and E2F levels significantly downregulated but not in a triple negative breast cancer model without this down regulation. However, no studies have been reported of [^18^F]FLT and CDK4/6 inhibitors in solid tumor models other than breast.

**Fig. 1:**
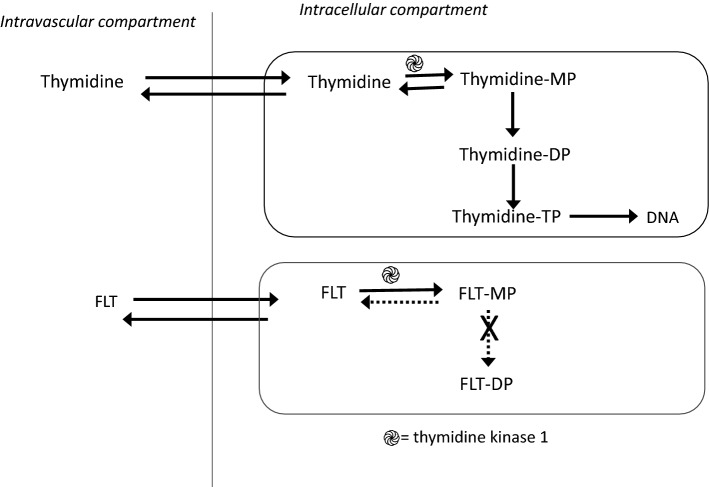
Uptake mechanism of thymidine and [^18^F]FLT. Like thymidine, FLT is transported across cell membranes by nucleotide transporters. After uptake, [^18^F]FLT is phosphorylated by TK 1 and trapped intracellularly. Incorporation into DNA is limited

The PET tracer [^18^F]FLT was developed as an imaging biomarker for one of cancer’s foundational characteristics proliferation. Unlike for [^18^F]FDG, [^18^F]FLT uptake is not a measure of general metabolism but instead reflects cell division activity. The mechanism of [^18^F]FLT uptake is well-established and occurs via nucleoside transporters. [8] Once intracellular, [^18^F]FLT is phosphorylated by thymidine kinase 1 (TK1), a cell cycle regulated enzyme and rate-limiting step of the thymidine salvage pathway. However, the [^18^F]FLT monophosphate cannot be further phosphorylated and incorporated into DNA and remains trapped in the cell. (Fig. 1). The [^18^F]FLT signal therefore reflects the enzymatic activity of TK1, which is linked to the cell cycle S phase.[9].

[^18^F]FLT uptake has been compared with gold standard histological proliferation markers like Ki-67 with good but imperfect correlation. [^18^F]FLT has also been used as an in vivo marker to predict response to therapy. The modulation of tracer uptake can be affected by complex cell cycle alterations, but taking these variables into account, [^18^F]FLT is a good indirect measure of proliferation, derived from the number of cells in S phase and the activity level of thymidine kinase. Interestingly, the correlations of these more direct measures of proliferation [9] support the hypothesis that [^18^F]FLT should provide a valid in vivo biomarker of the status of the D-CDK 4/6 pathway in tumors with on-target modulation by CKD 4/6 inhibitors since these inhibitors are very specific to the G1/S checkpoint without other effects on the cell cycle.

In this study we employed a well characterized patient derived xenograft (PDX) bladder model in a drug challenge that included the D-CDK 4/6 inhibitor palbociclib to determine if early modulation of the [^18^F]FLT uptake was correlated with tumor response in a non-breast cancer model.

## Materials and methods

### Animal model

Animal studies were performed according to the National Cancer Institute (NCI) at Frederick (Frederick, MD) Institutional Animal Care and Use Committee guidelines (IACUC Protocol No. 19-0008-B).

Tumor fragments (8 mm^3^) were harvested from mice bearing BL0382-F1232 (The Jackson Laboratory, Bar Harbor, ME) and directly implanted into the right flank of 6-week-old female NOD-*scid* gamma NSG mice weighing approximately 26 g (Frederick National Laboratory for Cancer Research, Biological Testing Branch Animal Production, Frederick MD). This model is a well characterized Patient Derived Xenograft (PDX) of a myo-invasive bladder cancer with no prior chemotherapy and with a partial loss of function p53 E177 mutation but functional Rb1. [10] A total of 24 mice were enrolled in the study when their tumor sizes were approximately centered at 250 mm^3^ (252 ± 134 mm^3^) and were randomized into four groups as noted below. There was no blinding.

### Drug therapy

**Fig. 2:**
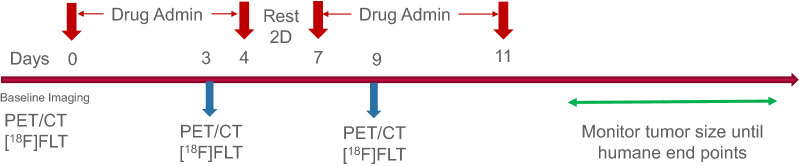
Study Protocol. Twenty-four NSG mice bearing patient derived xenografts of a well-characterized bladder tumor were randomized into 4 treatment groups: Control (n = 6); palbociclib (n = 6); temozolomide (n = 6); and palbociclib plus temozolomide (n = 6) and treated with two cycles of therapy or vehicle

The drug therapy protocol was based on a prior unpublished study that demonstrated tumor response with a combination therapy of palbociclib and temozolomide in this model. In addition to the combination therapy, we added single drug arms to determine single agent efficacy. When the mice met enrollment criteria based on tumor size they were randomized into 4 groups: control [n = 6]—vehicle (Klucel and 50 mM sodium lactate pH4); TMZ [n = 6]—temozolomide (50 mg/kg in Klucel); PALB [n = 6]—palbociclib (50 mg/kg in 50 mM sodium lactate pH4), and Combo [n = 6]—temozolomide (50 mg/kg) plus palbociclib (50 mg/kg) in Klucel and 50 mM sodium lactate pH4. The treatment regimen was 5 days therapy followed by 2 days rest and a second round of 5 days therapy. All drugs were administered PO once a day. Following the second cycle of therapy, drug administration was discontinued, and the mice were maintained until their tumors reached a size requiring humane termination (ACUC guidance > 2 cm in any linear dimension by caliper) or their clinical status required euthanasia. Figure 2 details the study design.

### Imaging

Xenograft tumor volume was calculated based on weekly caliper measurements. The volume was calculated from three dimensions measured. When the average tumor volume approached 250 mm^3^, baseline [^18^F]FLT positron emission tomography/computed tomography (PET/CT) imaging was initiated (NanoScan PET/CT, Mediso Medical Imaging Systems, Budapest, Hungary). [^18^F]FLT was prepared following the literature procedure [11]. The isolated radiochemical yields were in the range of 17–30% (n > 25, decay uncorrected) with a radiochemical purity > 99% and molar activity of 167–240 GBq/µmol.

### Animal preparation and handling

Standard imaging and animal handling protocols required maintaining the rodent’s temperature and anesthesia administration. The animal body temperature (thermostat controlled heated table at 34–37˚C) was maintained from the time the animal entered the imaging room, through anesthesia induction, imaging, and until recovery from anesthesia. Isoflurane anesthesia was administered via an induction chamber (3% pre-imaging) and nose cone (1.5–2% during imaging) with a carrier gas of oxygen at a flow of 1 l/min. Pulmonary function was monitored during scanning and the anesthesia (1.5–2% Isoflurane) was regulated to maintain a pulmonary rate between 50 and 90 breaths per minute (bpm).

### ***PET/CT ***Protocol

Mice were injected IV via the tail-vein with [^18^F]FLT (6.22 ± 0.75 MBq) at the planned time points. PET-imaging commenced at approximately 1-h post injection. Mice were imaged in the prone position for a 3-min CT for PET attenuation correction, followed by a 20-min PET acquisition. CT acquisition parameters were: 50 kVp, 980 μA, 300 ms per step, covering 360-degrees, helical scan, and pitch of 1. PET list-mode data were acquired using an energy window of 400–600 keV and a < 2 ns coincidence timing window. CT images were reconstructed using a cone beam algorithm resulting in 192 × 192 matrix and PET utilized Ordered Subset Maximum-Likelihood Expectation Maximization (OS ML-EM) with 4 iterations and 4 subsets, with corrections for attenuation (CT based Monte Carlo), decay, Randoms, scatter, and noise reduction regularization [12], resulting in a 0.4 mm^3^ voxel.

[^18^F]FLT PET/CT DICOM images were displayed and fused on a MIM workstation (v 6.6.5, MIM Software Inc, Cleveland, OH). Tumor [^18^F]FLT uptake was acquired from a volume of interest defined by CT, and the maximum standardized uptake value normalized by body weight (SUVbw max) was calculated using the same commercial software. Since reproducibility is critical with imaging at multiple time points, SUVmax was chosen rather than SUVmean because it is more reproducible, especially for smaller tumors [13, 14].

### Statistics

Values for each mouse (SUV bw max and tumor volumes) were normalized to 100% at day 0 (baseline); group mean and standard deviation were evaluated for each time point. A paired t-test was used to determine significance compared to baseline with p value for significance set to ≤ 0.05.

### Data availability

Data were generated by the authors and are included in the supplementary tables.

## Results

A significant decrease in [^18^F]FLT uptake in the palbociclib and combination arms was accompanied by a suppression of tumor growth during and for a time after therapy cessation. A significant increase in tumor size in the control and TMZ arms at each time point was accompanied by increasing [^18^F]FLT uptake.

The tumor size at enrollment was 252 ± 134 mm^3^. No mice were terminated during the therapy and up to day 26 after study initiation.

The data are summarized in Table 1.

**Table 1:** [^18^F]FLT SUVbw Max and Volume Changes during drug treatment

Treatment Day	Baseline	Day 3	P vs baseline	Day 9	p vs baseline
**SUV Max**					
Control	4.0 ± 2.4	5.1 ± 1.7	0.231	5.9 ± 1.9	0.0755
TMZ	4.1 ± 2.1	4.7 ± 3.1	0.521	6.1 ± 2.7	0.0418*
Palbociclib	6.0 ± 2.3	2.9 ± 2.2	0.042*	3.0 ± 1.0	0.0031*
Combo	6.1 ± 2.7	3.2 ± 1.7	0.011*	4.4 ± 2.2	0.0012*
**Volume**					
Control	234 ± 69	349 ± 163	0.0032*	442 ± 197	0.0008*
TMZ	291 ± 117	392 ± 117	0.0037*	512 ± 245	0.0076*
Palbociclib	207 ± 126	229 ± 125	0.2951	256 ± 155	0.4083
Combo	279 ± 209	269 ± 179	0.4316	301 ± 220	0.4151

Values are mean ± standard deviation

*Statistically significant

**Fig. 3:**
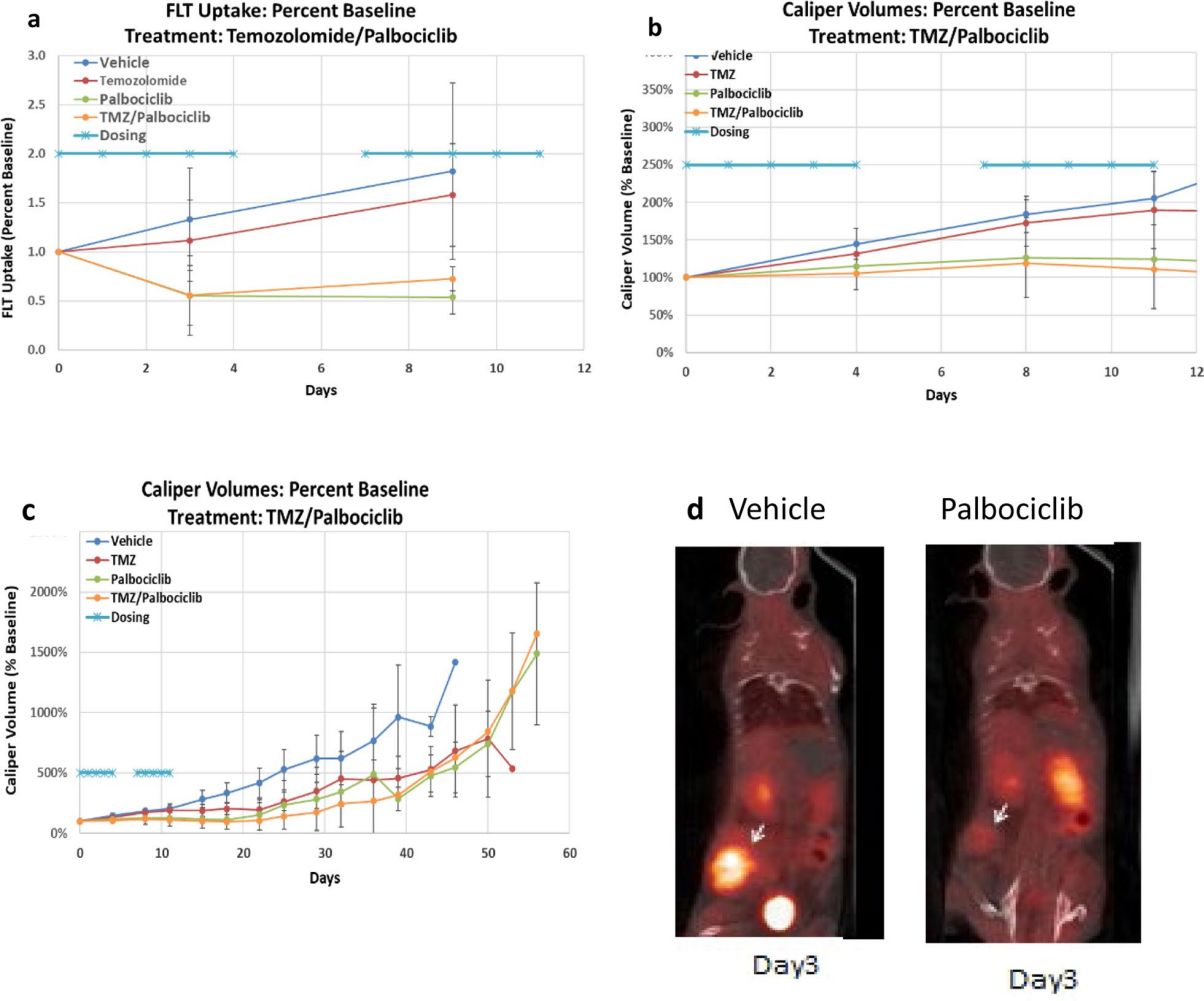
[^18^F]FLT uptake as SUVbw max and tumor volumes, normalized to baseline SUVbw max (3**a**) and caliper volumes (3**b** and 3**c**) are shown with respect to dosing (QDx5, rest 2 days, and repeat). Study and dosing were initiated at day 0. Plot 3**b** displays the caliper tumor volumes for the same study range as the PET/CT study for easy comparison while 3**c** displays the full study range until mice reach ACUC endpoint. Figure 3**d** shows typical images of control and palbociclib treated mice at day 3

At day three of therapy there was a statistically significant drop in [^18^F]FLT SUVbw max by 50% in both the palbociclib and combination therapy arms and uptake remained significantly depressed at day nine of therapy. [^18^F]FLT uptake in the TMZ and control arms increased but did not reach significance at day three. (Fig. 3a) [^18^F]FLT uptake continued to increase in both the TMZ and control arm at day nine with the TMZ arm reaching statical significance. The full SUV data set is presented in Additional file 1: Table S1 and is graphed in the Additional file 1: Figure S1.

Tumor volumes in the control group increased significantly by 45% and in the TMZ group by 31% in the first three days of treatment and continued to increase significantly at day nine. The tumor volumes in the palbociclib and combo groups did not significantly change at days three and nine compared to baseline (Fig. 3b). Following completion of the second cycle of treatment at day 11 tumor volumes continued to be measured until the tumors reached the ACUC limit of > 2 cm in any linear dimension or the animals exhibited signs of physical stress and were humanely terminated. Tumor volume remained stable from cessation of therapy until day 19 for the palbociclib arm and day 23 for the combo and TMZ arm. (Fig. 3c). The full tumor volume data set is presented in supplementary table S2 and is graphed in the Additional file 1: Figure S1. Figure 3d shows typical PET images for animals treated with control and with drug.

## Discussion

The concept of using rapid modulation of PET uptake as an in vivo pharmacodynamic assay is not new. Numerous preclinical studies using both [^18^F]FDG and [^18^F]FLT PET have shown modulation of signals on repeat imaging after therapeutic intervention in 24–72 h predictive of future response based on volume changes. [15].

Clinically it has been shown that when treating metastatic colon cancer lacking K-RAS mutation a nearly 50% decrease in [^18^F]FDG uptake at 48 h following an initial dose of panitumumab was predictive of response at 8 weeks of therapy [16].

In this study we investigated the early modulation (day 3 and day 9) of [^18^F]FLT PET after initiation of therapy in a well characterized [10] PDX model of invasive bladder cancer responsive to the CDK4/6 inhibitor palbociclib and palbociclib/temozolomide combination. While palbociclib and other CDK4/6 inhibitors have been approved by FDA in breast cancer in combination with endocrine therapy [3], our goal was to determine if [^18^F]FLT should be considered a potential in vivo biomarker for predictive response in other tumors since CDK4/6 inhibitors are increasingly being considered in other solid cancers either as single agents or in combination therapy [17, 18]. Preclinical data suggesting the utility of [^18^F]FLT imaging includes a recent publication that demonstrated early decrease in [^18^F]FLT PET in a breast cancer model using combination palbociclib/fulvestrant therapy [6] and in another breast cancer model with single agent therapy [7] However, no studies have been reported in non-breast cancer models.

**Fig. 4:**
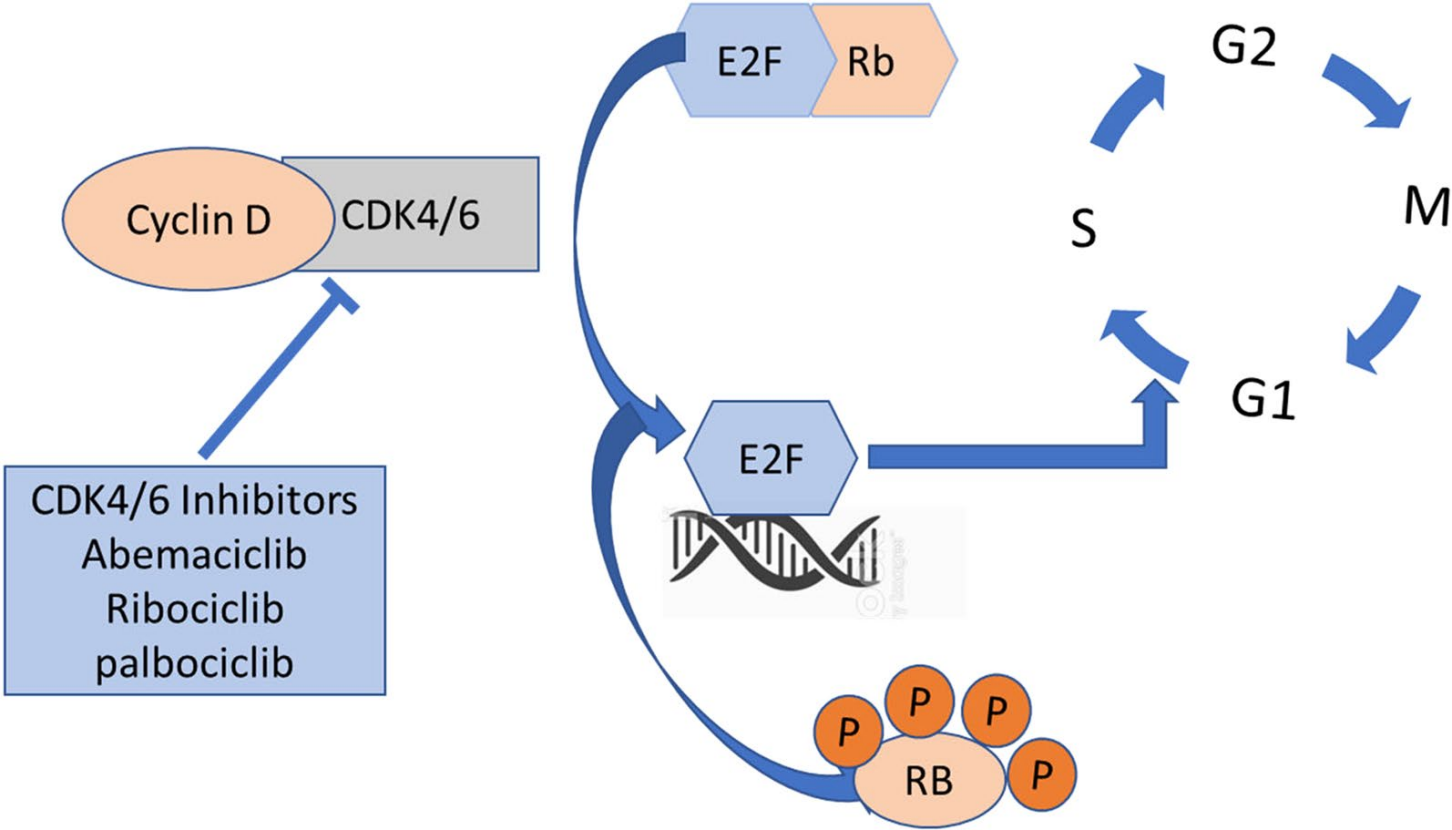
Under standard conditions CDK4/6, directed by upstream signaling, phosphorylates RB1 promoting E2F transcription and cell cycle progression from G1 to S. CDK4/6 inhibitors block this pathway, causing cells to stall at G1. This blockage in the cell cycle reduces uptake of thymidine and therefore of [^18^F]FLT

The CDK4/6 inhibitors act on the D-cyclin-dependent kinase 4/6-INK4-retinoblastoma (D-CDK 4/6- Rb) pathway, considered a key pathway regulating the G1-S phase transition in the cell cycle as shown in Fig. 4. Abnormal activation of this pathway has been reported in numerous cancer types. The retinoblastoma (RB) tumor suppressor regulates several processes associated with DNA repair and cell cycle progression [19]. RB suppresses the E2F family of genes that encode a collection of transcription factors involved in cell cycle regulation. Under standard conditions CDK4/6, directed by upstream signaling (PI3K/AKT/mTOR), phosphorylates RB1 promoting E2F transcription and cell cycle progression from G1 to S. While this pathway is important to DNA repair and genomic stability, it also is responsible for resistance to DNA-damaging therapeutics. In addition, this pathway is subject to abnormal deregulation of RB phosphorylation due to upregulated CDK4/6 from dysregulated input upstream. CDK4/6 inhibitors block the phosphorylation of RB1 and lead to G1 arrest [17].

[^18^F]FLT is a well-established PET imaging probe that reflects the status of cellular proliferation and has long been applied in oncology studies [20]. In a systematic review, Schelhass and co-authors found 102 papers investigating the correlation of [^18^F]FLT uptake to histological proliferation markers (mostly Ki67) and demonstrated correlation in the vast majority of studies [21]. Uptake of [^18^F]FLT occurs via nucleoside transporters notably nucleoside transporter 1 (hENT1) and once intracellular, it is phosphorylated by thymidine kinase 1 (TK1), a cell cycle regulated enzyme and rate-limiting step of the thymidine salvage pathway [22]. The [^18^F]FLT signal is therefore a measure of the enzymatic activity of TK1. Tumor accumulation of [^18^F]FLT strongly correlates with TK1 activity and percentage of cells in S phase [23, 24].

[^18^F]FLT PET should not be confused with [^18^F]FDG PET which measures aerobic glycolysis and correlates with tumor glycolytic reprogramming of the tumor metabolome. Rather [^18^F]FLT is a marker of DNA synthesis and reports on the status of the cell cycle especially correlated to S phase. [^18^F]FLT imaging in conjunction with a specific inhibitor of G1/S that rapidly down regulates TK1 and depletes S phase provides an in vivo measure of on target effect prior to measurable tumor volume change. Arrest of cells at G1 will cause a reduction in uptake of [^18^F]FLT compared to cells that are cycling normally. [^18^F]FLT imaging for prediction of therapeutic response in drug treatment models has shown a significant reduction in [^18^F]FLT uptake versus baseline as early as 24 h following initiation of therapy and has consistently shown reductions at 72 h [15]. Pre-clinical studies have shown that CDK4/6 inhibitor therapy leads to halt of G1/S, and the cells in S phase pass through G2/M with rapid washout. If the G1/S halt is persistent then cells will die primarily through senescence depending on p53 driven autophagy [24].

In this study we observed a significant reduction in [^18^F]FLT at both 3 and 9 days following initiation of therapy in both the palbociclib and palbociclib plus temozolomide therapy arms. During this time and persisting out to 19 days (12 days post end of therapy) there was significant suppression of tumor growth in these two groups. In the control group, we observed progressive growth and corresponding increase in [^18^F]FLT PET uptake. In the temozolomide group there was initially progressive growth as in the control arm with increased [^18^F]FLT uptake from day 3 to 9. By day 23, following cessation of therapy, all groups demonstrated progressive growth. Clearly the single agent palbociclib was driving the therapeutic response, consistent with the early [^18^F]FLT measures. On the other hand, temozolomide as a single agent was not effective and [^18^F]FLT uptake increased in line with control at day 3 and 9, as has been reported in a glioma study with TMZ resistant cells [26]. The fact that the combination therapy arm had prolonged suppression of growth over the palbociclib alone arm following cessation of therapy is interesting and warrants further investigation.

Our study is limited in that this was a single well characterized PDX model in a small number of animals. However, preclinical efficacy data has been reported [5] in other models, both responsive and non-responsive to CDK4/6 inhibitors, suggesting that further studies of [^18^F]FLT for response assessment to aid in development of this class of compounds in tumors other than breast is warranted. While [^18^F]FLT is not currently commercially available for clinical studies, it has been implemented in numerous clinical trials under active INDs in many institutions.

## Conclusion

In this exploratory study we demonstrated that early decreased uptake of [^18^F]FLT PET occurred with suppression of tumor growth in a PDX model of invasive bladder disease treated with palbociclib or palbociclib/temozolomide. Temozolomide as a single agent was not effective and early increased [^18^F]FLT uptake was consistent with lack of therapeutic response. Based on these findings and supported by referenced preclinical publications, it appears [^18^F]FLT may serve as an in vivo biomarker for CDK4/6 inhibitor therapy in solid tumor histologies beyond breast. Subsequent studies are needed to confirm these results and to determine if other tumor types responsive to CDK4/6 inhibitors also demonstrate similar modulation of [^18^F]FLT uptake.

## Supplementary Information

**Additional file 1: Figure S1**. Mean and standard deviation [18F]FLT SUVbw max) (**A**) and caliper measured tumor volumes (**B**) for individual mice in each cohort at each timepoint and normalized to baseline. The red horizontal line signifies the normalized baseline for easy comparison. **Table S1.** Individual animal data for [^18^F]FLT uptake [SUVbw max]. **Table S2.** Individual animal data for tumor volume (mm^3^).

## Data Availability

All data generated or analyzed during this study are included in this published article [and its supplementary information files].

## Abbreviations

[^18^F]FLT: [^18^F]fluoro-3'-deoxythymidine
CDK4/6: Cyclin-dependent kinase 4/6-INK4-retinoblastoma
CT: X-ray computed tomography
E2F: Genes encoding a family of transcription factors regulating cell cycle
ER: Estrogen receptor
ERBB2: Receptor tyrosine-protein kinase erbB-2
HER2: Human epidermal growth factor receptor 2
PDX: Patient derived xenograft
PET: Positron Emission Tomography
pRB, Rb, Rb1: Retinoblastoma proteins
SUVbw max: Maximum standardized uptake value normalized by body weight
TK1: Thymidine kinase 1
TMZ: Temozolomide
