# Supplementary material for: 3'-[18F]fluoro-3'-deoxythymidine ([18F]FLT) Positron Emission Tomography as an In Vivo Biomarker of inhibition of CDK 4/6-Rb pathway by Palbociclib in a patient derived bladder tumor
Source: J Transl Med. 2022 Aug 18;20:375. doi: 10.1186/s12967-022-03580-8 (PMC9389794; doi:10.1186/s12967-022-03580-8)
Supplement: Supplementary file 1 — Additional file 1: Figure S1. Mean and standard deviation [18F]FLT SUVbw max) (A) and caliper measured tumor volumes (B) for individual mice in each cohort at each timepoint and normalized to baseline. The red horizontal line signifies the normalized baseline for easy comparison. Table S1. Individual animal data for [18F]FLT uptake [SUVbw max]. Table S2. Individual animal data for tumor volume (mm3). [file 12967_2022_3580_MOESM1_ESM.docx]

**SUPPLEMENTARY DATA**

Supplementary figure


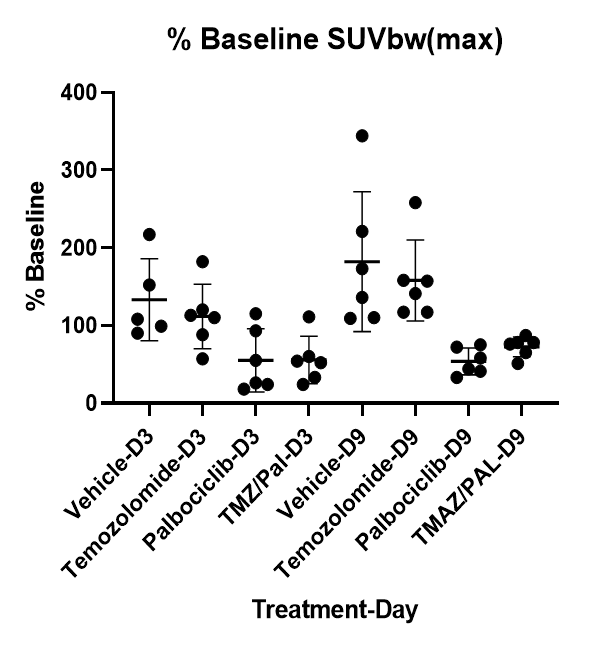

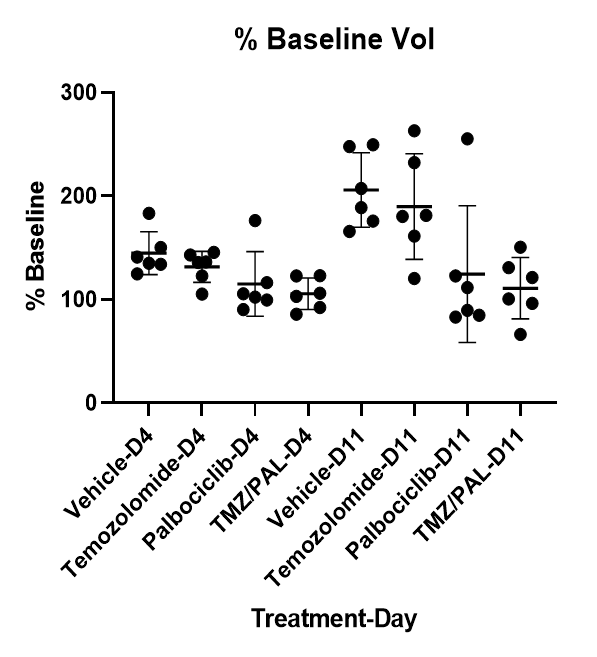


**A**

**B**

Figure S1: Mean and standard deviation [18F]FLT SUVbw max) (A) and caliper measured tumor volumes (B) for individual mice in each cohort at each timepoint and normalized to baseline.  The red horizontal line signifies the normalized baseline for easy comparison.

Table S1. Individual animal data for [^18^F]FLT uptake [SUVbw max]

|  |  | **SUV Max** | | | **% Baseline** | | |
| --- | --- | --- | --- | --- | --- | --- | --- |
|  | **Treatment Day** | **0** | **3** | **9** | **0** | **3** | **9** |
| **M#** | **Group** |  |  |  |  |  |  |
| 1812 | Vehicle | 4.32 | 3.89 | 4.72 | 100% | 90% | 109% |
| 1813 | Vehicle | 6.24 | 6.74 | 8.45 | 100% | 108% | 136% |
| 1816 | Vehicle | 7.34 | 7.26 | 8.08 | 100% | 99% | 110% |
| 1818 | Vehicle | 2.53 | 3.84 | 4.37 | 100% | 152% | 173% |
| 1822 | Vehicle | 1.70 | NA | 5.85 | 100% | NA | 344% |
| 1823 | Vehicle | 1.84 | 4.00 | 4.08 | 100% | 217% | 221% |
| 1808 | Temozolomide | 8.38 | 10.07 | 9.81 | 100% | 120% | 117% |
| 1819 | Temozolomide | 2.92 | 1.66 | 4.12 | 100% | 57% | 141% |
| 1820 | Temozolomide | 3.12 | 2.73 | 4.90 | 100% | 88% | 157% |
| 1826 | Temozolomide | 3.59 | 4.07 | 4.19 | 100% | 113% | 117% |
| 1829 | Temozolomide | 2.76 | 3.03 | 4.35 | 100% | 110% | 158% |
| 1832 | Temozolomide | 3.67 | 6.67 | 9.46 | 100% | 182% | 258% |
| 1804 | Palbociclib | 5.84 | 1.07 | 1.95 | 100% | 18% | 33% |
| 1805 | Palbociclib | 4.10 | 2.26 | 2.96 | 100% | 55% | 72% |
| 1806 | Palbociclib | 7.58 | 1.95 | 3.31 | 100% | 26% | 44% |
| 1817 | Palbociclib | 8.34 | 1.98 | 3.40 | 100% | 24% | 41% |
| 1827 | Palbociclib | 2.59 | 2.97 | 1.93 | 100% | 115% | 75% |
| 1834 | Palbociclib | 7.80 | 7.25 | 4.51 | 100% | 93% | 58% |
| 1802 | TMZ/Palbociclib | 10.85 | 5.65 | 8.51 | 100% | 52% | 78% |
| 1803 | TMZ/Palbociclib | 5.14 | 2.75 | 2.63 | 100% | 54% | 51% |
| 1807 | TMZ/Palbociclib | 6.81 | 1.60 | 4.42 | 100% | 24% | 65% |
| 1814 | TMZ/Palbociclib | 6.53 | 3.94 | 4.94 | 100% | 60% | 76% |
| 1824 | TMZ/Palbociclib | 3.71 | 4.11 | 2.88 | 100% | 111% | 78% |
| 1825 | TMZ/Palbociclib | 3.60 | 1.18 | 3.13 | 100% | 33% | 87% |

Table S2: individual animal data for tumor volume (mm^3^)

|  |  | **Study Days** | | | | | | | | | | | | | | | | |
| --- | --- | --- | --- | --- | --- | --- | --- | --- | --- | --- | --- | --- | --- | --- | --- | --- | --- | --- |
| **Group** | **Mouse ID** | **0** | **4** | **8** | **11** | **15** | **18** | **22** | **25** | **29** | **32** | **36** | **39** | **43** | **46** | **50** | **54** | **57** |
| Group 1 | 1812 | 184 | 230 | 306 | 305 | 362 | 395 | 452 | 526 | 705 | 727 | 882 | 1110 | 1738 | 2616 |  |  |  |
| Vehicle TMZ/ Vehicle Palbociclib | 1818 | 212 | 319 | 426 | 373 | 588 | 673 | 800 | 1053 | 1460 | 1626 | 1868 | 2602 |  |  |  |  |  |
|  | 1823 | 368 | 673 | 829 | 910 | 1430 | 1658 | 2021 | 2483 |  |  |  |  |  |  |  |  |  |
|  | 1813 | 240 | 323 | 420 | 452 | 513 | 635 | 815 | 969 | 1123 | 1135 | 1375 | 1422 | 1982 |  |  |  |  |
|  | 1816 | 215 | 288 | 376 | 536 | 630 | 738 | 943 | 1239 | 1462 | 1832 | 2453 | 3070 |  |  |  |  |  |
|  | 1822 | 184 | 259 | 296 | 381 | 600 | 738 | 1019 | 1339 | 1601 |  |  |  |  |  |  |  |  |
| Group 1: % of baseline | 1812 | 100% | 125% | 166% | 166% | 197% | 214% | 245% | 285% | 383% | 395% | 479% | 602% | 943% | 1419% |  |  |  |
|  | 1818 | 100% | 150% | 200% | 176% | 277% | 317% | 377% | 496% | 687% | 766% | 880% | 1225% |  |  |  |  |  |
|  | 1823 | 100% | 183% | 226% | 248% | 389% | 451% | 550% | 676% |  |  |  |  |  |  |  |  |  |
|  | 1813 | 100% | 135% | 175% | 189% | 214% | 265% | 340% | 404% | 468% | 474% | 574% | 593% | 827% |  |  |  |  |
|  | 1816 | 100% | 134% | 175% | 249% | 293% | 343% | 439% | 576% | 680% | 852% | 1140% | 1427% |  |  |  |  |  |
|  | 1822 | 100% | 141% | 161% | 207% | 326% | 402% | 554% | 729% | 871% |  |  |  |  |  |  |  |  |
| Group 1 | Mean | 100% | 145% | 184% | 206% | 283% | 332% | 417% | 528% | 618% | 621% | 768% | 962% | 885% | 1419% |  |  |  |
|  | SD | 0% | 21% | 24% | 36% | 71% | 87% | 122% | 167% | 194% | 221% | 302% | 429% | 82% |  |  |  |  |
| Group 2: TMZ | 1826 | 392 | 559 | 627 | 706 | 666 | 711 | 766 | 1093 | 1588 | 1930 |  |  |  |  |  |  |  |
|  | 1829 | 218 | 317 | 485 | 574 | 548 | 613 | 654 | 816 | 1277 | 1859 |  |  |  |  |  |  |  |
|  | 1832 | 227 | 279 | 343 | 273 | 234 | 301 | 289 | 374 | 555 | 732 | 1320 |  |  |  |  |  |  |
|  | 1808 | 160 | 168 | 234 | 289 | 288 | 331 | 295 | 453 | 586 | 802 | 908 | 1054 | 1153 | 1523 | 1801 |  |  |
|  | 1819 | 287 | 389 | 448 | 463 | 584 | 570 | 494 | 807 | 873 | 1011 | 1115 | 1145 | 1514 |  |  |  |  |
|  | 1820 | 468 | 637 | 933 | 1087 | 998 | 1030 | 912 | 886 | 850 | 850 | 1063 | 1441 | 1584 | 1923 | 2056 | 2512 |  |
| Group 2: % of baseline | 1826 | 100% | 143% | 160% | 180% | 170% | 181% | 196% | 279% | 405% | 493% |  |  |  |  |  |  |  |
|  | 1829 | 100% | 145% | 222% | 263% | 251% | 281% | 299% | 374% | 585% | 852% |  |  |  |  |  |  |  |
|  | 1832 | 100% | 123% | 151% | 120% | 103% | 133% | 127% | 165% | 244% | 322% | 581% |  |  |  |  |  |  |
|  | 1808 | 100% | 105% | 146% | 181% | 180% | 207% | 185% | 284% | 367% | 501% | 568% | 659% | 721% | 952% | 1127% |  |  |
|  | 1819 | 100% | 136% | 156% | 161% | 203% | 199% | 172% | 281% | 304% | 352% | 388% | 399% | 528% |  |  |  |  |
|  | 1820 | 100% | 136% | 199% | 232% | 213% | 220% | 195% | 189% | 181% | 182% | 227% | 308% | 338% | 411% | 439% | 537% |  |
| Group 2 | Mean | 100% | 131% | 172% | 190% | 187% | 203% | 196% | 262% | 348% | 450% | 441% | 455% | 529% | 682% | 783% | 537% |  |
|  | SD | 0% | 15% | 31% | 51% | 50% | 49% | 57% | 75% | 142% | 230% | 168% | 182% | 192% | 383% | 486% |  |  |
| Group 3 Palbociclib | 1804 | 101 | 103 | 106 | 85 | 93 | 74 | 97 | 196 | 238 | 337 | 392 | 434 | 739 | 866 | 1155 | 1604 | 1948 |
|  | 1806 | 394 | 391 | 428 | 352 | 294 | 289 | 342 | 488 | 628 | 724 | 824 | 843 | 1219 | 1398 | 1902 |  |  |
|  | 1817 | 159 | 144 | 132 | 132 | 109 | 109 | 119 | 132 | 184 | 233 | 238 | 278 | 532 | 619 | 917 | 1033 | 1308 |
|  | 1805 | 105 | 122 | 113 | 117 | 85 | 82 | 132 | 204 | 225 | 271 | 325 | 333 | 571 | 681 | 936 | 1357 | 1803 |
|  | 1827 | 338 | 356 | 413 | 414 | 413 | 372 | 432 | 557 | 535 | 736 | 930 | 968 | 1552 | 1609 | 2058 |  |  |
|  | 1834 | 148 | 260 | 343 | 377 | 380 | 396 | 597 | 945 | 1198 | 1366 | 2359 |  |  |  |  |  |  |
| Group 3:% of baseline | 1804 | 100% | 102% | 105% | 85% | 92% | 73% | 96% | 194% | 235% | 334% | 388% | 429% | 731% | 857% | 1142% | 1587% | 1927% |
|  | 1806 | 100% | 99% | 109% | 89% | 75% | 73% | 87% | 124% | 159% | 184% | 209% | 214% | 309% | 355% | 483% |  |  |
|  | 1817 | 100% | 90% | 83% | 83% | 68% | 69% | 75% | 83% | 115% | 146% | 150% | 174% | 334% | 388% | 576% | 648% | 821% |
|  | 1805 | 100% | 116% | 108% | 111% | 81% | 78% | 126% | 194% | 214% | 258% | 310% | 317% | 544% | 649% | 892% | 1293% | 1718% |
|  | 1827 | 100% | 105% | 122% | 123% | 122% | 110% | 128% | 165% | 158% | 218% | 275% | 287% | 460% | 476% | 609% |  |  |
|  | 1834 | 100% | 176% | 232% | 255% | 257% | 268% | 404% | 640% | 812% | 925% | 1598% |  |  |  |  |  |  |
| Group 3 | Mean | 100% | 115% | 126% | 124% | 116% | 112% | 153% | 233% | 282% | 344% | 488% | 284% | 476% | 545% | 740% | 1176% | 1489% |
|  | SD | 0% | 31% | 53% | 66% | 72% | 78% | 125% | 204% | 263% | 292% | 550% | 99% | 172% | 208% | 272% | 480% | 588% |
| Group 4 TMZ/ Palbociclib | 1803 | 196 | 181 | 192 | 188 | 142 | 141 | 141 | 150 | 207 | 270 | 280 | 322 | 524 | 608 | 926 | 1060 | 1337 |
|  | 1807 | 134 | 164 | 164 | 162 | 142 | 125 | 147 | 193 | 297 | 478 | 523 | 628 | 890 | 1177 | 1463 | 1658 | 2195 |
|  | 1824 | 439 | 465 | 667 | 573 | 583 | 687 | 671 | 609 | 808 | 1052 | 1169 | 1428 | 2054 |  |  |  |  |
|  | 1802 | 167 | 172 | 175 | 168 | 147 | 119 | 115 | 187 | 223 | 337 | 379 | 461 | 781 | 874 | 1209 | 1431 | 2006 |
|  | 1814 | 618 | 529 | 486 | 408 | 347 | 311 | 359 | 426 | 693 | 757 | 847 | 1208 | 1473 | 1516 | 1962 |  |  |
|  | 1825 | 86 | 106 | 135 | 129 | 117 | 112 | 150 | 257 | 245 | 350 | 376 | 415 | 817 | 1012 | 1374 | 1803 | 2660 |
| Group 4:  % of baseline | 1803 | 100% | 92% | 98% | 96% | 73% | 72% | 72% | 77% | 106% | 138% | 143% | 164% | 267% | 310% | 472% | 541% | 682% |
|  | 1807 | 100% | 123% | 123% | 121% | 106% | 94% | 110% | 145% | 222% | 358% | 391% | 470% | 666% | 881% | 1096% | 1242% | 1644% |
|  | 1824 | 100% | 106% | 152% | 131% | 133% | 156% | 153% | 139% | 184% | 239% | 266% | 325% | 467% |  |  |  |  |
|  | 1802 | 100% | 103% | 104% | 100% | 88% | 71% | 69% | 112% | 133% | 201% | 226% | 275% | 466% | 522% | 722% | 855% | 1198% |
|  | 1814 | 100% | 86% | 79% | 66% | 56% | 50% | 58% | 69% | 112% | 123% | 137% | 196% | 239% | 246% | 318% |  |  |
|  | 1825 | 100% | 123% | 157% | 150% | 136% | 131% | 174% | 299% | 285% | 407% | 437% | 483% | 949% | 1176% | 1597% | 2096% | 3092% |
| Group 4 | Mean | 100% | 105% | 119% | 111% | 99% | 96% | 106% | 140% | 174% | 244% | 267% | 319% | 509% | 627% | 841% | 1183% | 1654% |
|  | SD | 0% | 15% | 31% | 30% | 32% | 40% | 48% | 84% | 71% | 116% | 125% | 135% | 266% | 395% | 515% | 673% | 1036% |

Blank cells indicate the mouse was euthanized at the prior time point.
